# *Lect2* deficiency is characterised by altered cytokine levels and promotion of intestinal tumourigenesis

**DOI:** 10.18632/oncotarget.26335

**Published:** 2018-11-23

**Authors:** Kirsty R. Greenow, Matthew Zverev, Stephanie May, Howard Kendrick, Geraint T. Williams, Toby Phesse, Lee Parry

**Affiliations:** ^1^ European Cancer Stem Cell Research Institute, Cardiff School of Biosciences, Cardiff University, Cardiff, UK; ^2^ School of Medicine, Cardiff University, Cardiff, UK

**Keywords:** Lect2, Wnt, colorectal cancer, inflammation, intestine

## Abstract

Leukocyte cell-derived chemotaxin 2 (Lect2) is a chemokine-like chemotactic factor that has been identified as a downstream target of the Wnt signalling pathway. Whilst the primary function of Lect2 is thought to be in modulating the inflammatory process, it has recently been implicated as a potential inhibitor of the Wnt pathway. Deregulation of the Wnt pathway, often due to loss of the negative regulator *APC*, is found in ~80% of colorectal cancer (CRC). Here we have used the *Apc^Min/+^Lect2^−/−^* mouse model to characterise the role of Lect2 in Wnt-driven intestinal tumourigenesis. Histopathological, immunohistochemical, PCR and flow cytometry analysis were employed to identify the role of Lect2 in the intestine. The *Apc^Min/+^Lect2^−/−^* mice had a reduced mean survival and a significantly increased number of adenomas in the small intestine with increased severity. Analysis of *Lect2* loss indicated it had no effect on the Wnt pathway in the intestine but significant differences were observed in circulating inflammatory markers, CD4+ T cells, and T cell lineage-specification factors. In summary, in the murine intestine loss of Lect2 promotes the initiation and progression of Wnt-driven colorectal cancer. This protection is performed independently of the Wnt signalling pathway and is associated with an altered inflammatory environment during Wnt-driven tumorigenesis.

## INTRODUCTION

The progression of colorectal cancer (CRC) from a benign to a malignant state is a multi-stage process that requires key changes in both oncogenes and tumour suppressor genes. A critical pathway in the pathogenesis of CRC is the Wnt signaling pathway and inactivation of *APC* or activating mutations in β-catenin are found in the majority of patients presenting with CRC [1]. It is therefore not surprising that the Wnt pathway and its downstream mediators are attractive targets for new therapeutics and several small molecule inhibitors and natural compounds have been identified to have potential therapeutic value against Wnt-driven tumorigenesis through either direct or indirect mechanisms [2].

Leukocyte cell-derived chemotaxin 2 (Lect2) is a chemokine-like chemotactic factor that has been identified as a downstream target of the Wnt signalling pathway [3]. Lect2 has a key role in several pathological conditions including rheumatoid arthritis [4, 5], renal amyloidosis [6], hepatocellular carcinoma [3, 7], liver injury [5] and sepsis [8], where its main activity is thought to be in modulating the inflammatory response. In the liver, Lect2 has a protective anti-inflammatory role in β-catenin-induced tumorigenesis and loss of this chemokine results in tumour progression and metastatic disease [3]. Previous studies have implicated Lect2 as a potential inhibitor of the Wnt pathway and Lect2 has been hypothesised to play a key role in the inhibition of intestinal tumorigenesis observed in the *Apc^Min/+^Mbd2^−/−^* mouse model due to this inhibitory effect on Wnt signalling [4]. Whilst the precise function and mechanism of Lect2 in the development of CRC is still unclear, the potential of this molecule as a regulator of the Wnt pathway warrants further investigation. In addition, the role of Lect2 in inflammation and the potential of this chemokine to affect intestinal tumour development by altering the inflammatory response is of significant interest and may aid the identification of novel targets in the treatment of this disease.

Therefore, to investigate the role of Lect2 in Wnt-driven intestinal tumorigenesis, we generated an *Apc^Min/+^Lect2^−/−^* mouse model. Our study demonstrates that loss of Lect2 in the *Apc^Min/+^* mouse had a significant pro-tumorigenic effect, confirming a protective tumour suppressor role for Lect2 in Wnt-driven CRC.

## RESULTS

### Loss of *Lect2* modifies Wnt-driven tumourigenesis and reduces survival

**Figure 1:**
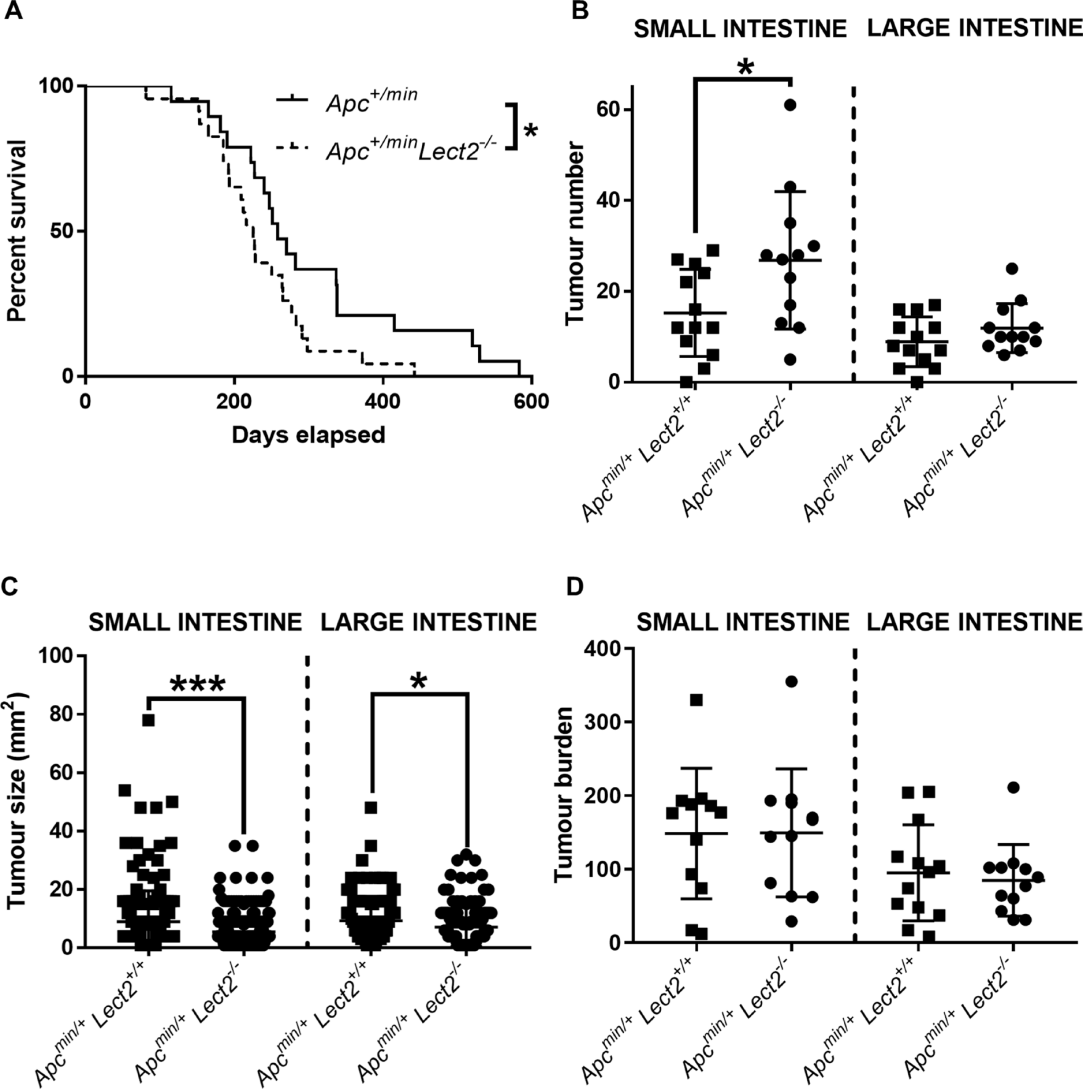
Homozygous Lect2 deletion drives Wnt-driven tumorigenesis and decreases survival (**A**) Kaplan–Meier survival analysis indicating a decrease in survival of the experimental *Apc^+/min^Lect2^−/−^* (*N* = 23) mice compared to the control *Apc^+/min^Lect2^+/+^* (*N* = 19) cohort (Log-rank (Mantel-Cox) test, *P* = 0.042). Formalin fixed tissue was used to quantify small (SI) and large (LI) intestine tumour number (**B**), size (**C**) and burden (**D**) at time of death. Indicating an increase in number of small intestine tumours in *Apc^min/+^Lect2^−/−^* mice (B; Mann–Whitney *P* = 0.0138) with a corresponding decrease in tumour size in the small (*P* = 0.0001) and large (*P* = 0.0038) intestine (C). Overall burden was unaltered at death (D) due to the increase in number of tumours compensating for their reduced size due to reduced longevity.

Lect2 has been implicated as a novel Wnt repressor and a potential tumour suppressor in CRC [4]. In order to test this hypothesis we crossed the *Lect2^−/−^* allele [5] onto an *Apc^Min/+^* background. The *Apc^Min/+^* mouse model is a well-established CRC model that is heterozygous for a mutation in the *Apc* gene and develops multiple intestinal neoplasia. Cohorts of at least 15 experimental and control mice were aged and the mice were monitored regularly for signs of intestinal tumours (rectal bleeding, prolapse, anaemia) or other illness and were taken for analysis when they became symptomatic of disease. Comparison of the endpoint demonstrated the mean survival of *Apc^Min/+^Lect2^−/−^* (239 days; *N* = 23) was significantly shorter than in the control *Apc^Min/+^Lect2^+/+^* (308 days; *N* = 19) cohort (Log-rank (Mantel-Cox) test, *P* = 0.042) (Figure 1A). All cohorts developed adenomas within the small intestine and the large intestine, with no other *Apc^+/min^* associated clinical phenotypes observed. The decrease in survival of *Apc^Min/+^Lect2^−/−^* mice correlated with a significantly increased number of adenomas in the small intestine compared to the *Apc^Min/+^Lect2^+/+^* mice at death (mean of 26.8 tumours versus 15.2 tumours, Mann–Whitney *U*-test, *P* = 0.0138; Figure 1B). No significant difference was seen in the number of adenomas in the large intestine. By contrast, a significant reduction in mean tumour size was observed in the *Apc^Min/+^Lect2^−/−^* cohort, both in the small (5.6 mm^2^ versus 8.9 mm^2^, Mann–Whitney *U*-test, *P* = 0.0001) and large (7.1 mm^2^ versus 9.2 mm^2^, Mann–Whitney *U*-test, *P* = 0.0038) intestine (Figure 1C). As further analysis of intestinal tumour burden at survival endpoint (Figure 1D) indicated no significant difference between cohorts the reduction of mean tumour size in the *Apc^Min/+^Lect2^−/−^* is likely a reflection of the animals’ shortened longevity.

As we previously identified upregulation of *Lect2* the tumour suppression observed in the *Apc^Min/+^Mbd2^−/−^* [9] we generated *Apc^Min/+^Mbd2^−/−^Lect2^−/−^* to clarify its role in this phenotype. However, the additional loss of *Lect2* failed to reverse the suppression of tumour initiation observed in the *Apc^Min/+^Mbd2^−/−^* mice (Supplementary Figure 1A and 1B) indicating *Lect2* does not play a significant role in the suppression of intestinal tumourigenesis observed in that model.

In summary this data indicates that *Lect2* plays a role in preventing tumour initiation in the *Apc^Min/+^*intestine.

### *Lect2* status influences tumour severity in the small intestine

**Figure 2:**
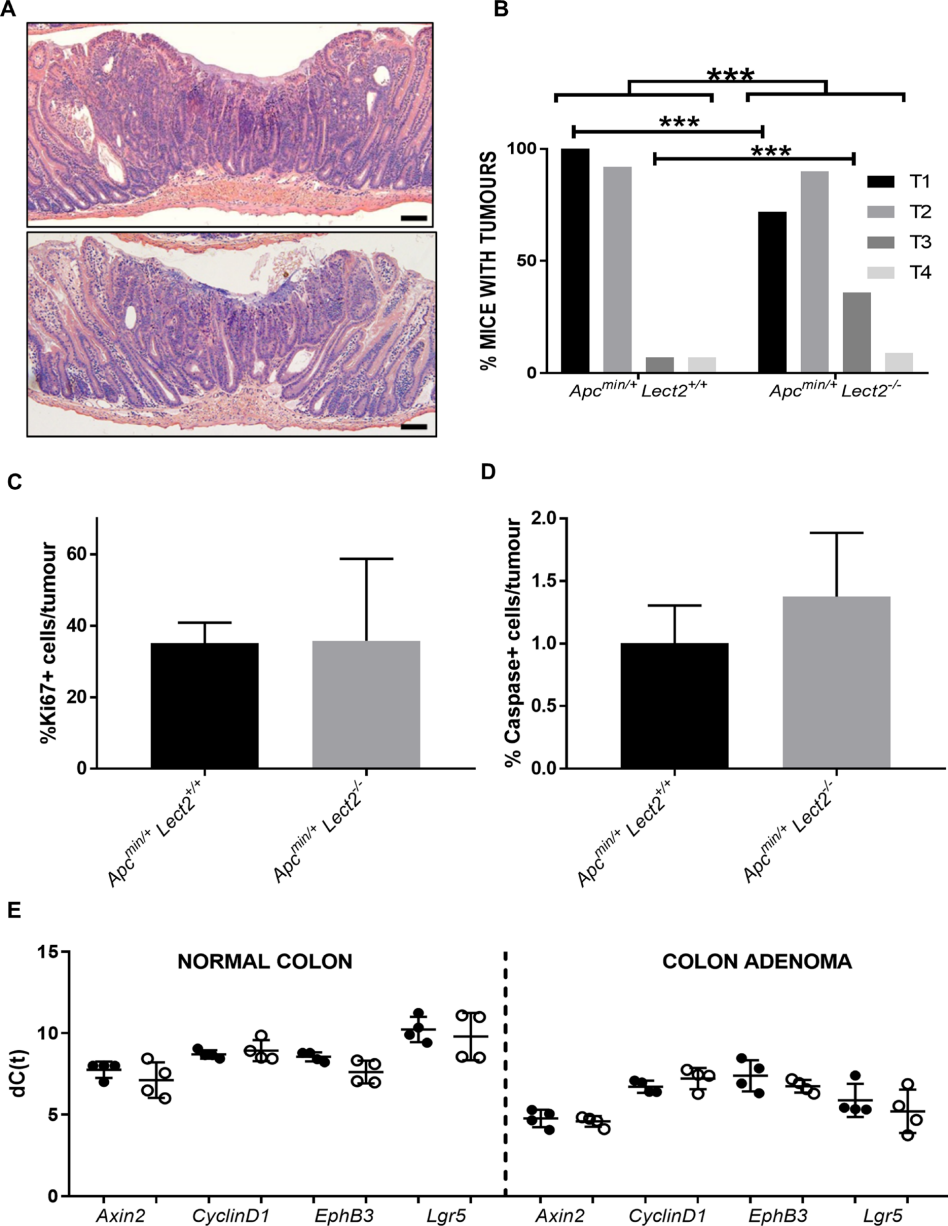
Loss of *Lect2* accelerates Wnt-activated tumour progression Histological examination of small intestine adenoma tumours from both *Apc*^Min/+^*Lect2*^+/+^ (**A**; top panel) and *Apc^Min/+^Lect2*^−/−^ (A; bottom panel) mice (Bar 50 μm). (**B**) Classification of tumour grades from *Apc^Min/+^Lect2*^+/+^ (*N* = 19) and *Apc^Min/+^Lect2*^−/−^ (*N* = 23) mice. Demonstrating an increase in overall severity upon *Lect2* loss (Chi-Squared Test P<0.0001) with a decrease in T1 and increase in T3 lesions (Chi-Squared Test *P* < 0.0001). Tissue sections from *Apc^Min/+^Lect2*^+/+^ (*N* = 4) and *Apc^Min/+^Lect2*^+/+^ (*N* = 4) mice were stained with antibodies detecting proliferation marker Ki67 (**C**) and apoptosis marker cleaved-Caspase 3 (**D**). Quantification by scoring positively-stained cells in ten fields of view per tumour indicated no significant changes. (**E**) qRT-PCR analysis of down-stream Wnt-targets from normal colon and and an adenoma from *Apc^Min/+^Lect2*^+/+^ (●) and *Apc^Min/+^Lect2*^+/+^ (○) mice. The data shown is in terms of the dC(t) values used to calculate the ddC(t) fold-change values.

To gain a clearer understanding of the decrease in survival seen in the *Apc^Min/+^Lect2^−/−^* cohort and to fully characterise the effect of *Lect2* loss on the *Apc^Min/+^* phenotype we histologically characterised the small intestine and the large intestine tumours of both the *Apc^Min/+^Lect2^+/+^* and the *Apc^Min/+^Lect2^−/−^* cohorts (Figure 2A). Tumours were classified as microadenomas (T1); adenomas (T2); early invasive adenocarcinomas showing invasion into the submucosa but not the muscularis propria (T3); and advanced invasive adenocarcinoma that penetrated into or through the muscularis propria (T4). The control (*Apc^Min/+^Lect2^+/+^*) mice had benign microadenomas T1 (Figure 2A (top panel); *n* = 13/13; 100%) and T2 adenomas (*n* = 12/13; 92.3%), with rare T3 adenocarcinomas (*n* = 1/13; 7.7%) or T4 tumours invading into the muscularis propria (*n* = 1/13; 7.7%; Figure 2B). The *Apc^Min/+^Lect2^−/−^* displayed a significant shift in progression with a reduction in the percentage of mice with early T1 lesions to 72.8% (*n* = 8/11) with a corresponding increase in early invasive T3 lesions to 36.4% (*n* = 4/11) (Chi-Squared Test *P* < 0.0001; Figure 2A (bottom panel) and 2B). To understand whether progression was due to alteration in cell homeostasis we next performed immunohistochemical analysis for Ki67, a marker of proliferation, and cleaved Caspase-3, a marker of apoptosis. The percentage of positively-staining cells in tumours of both the *Apc^Min/+^Lect2^+/+^* (*N* = 4) and *Apc^Min/+^Lect2^−/−^* (*N* = 5) cohorts indicated that loss of Lect2 had no significant effect on either cell proliferation (Figure 2C) or cell death (Figure 2D) in our Wnt-driven tumours. To address the Wnt-inhibitory function of *Lect2* we used qRT-PCR analysis to compare the expression of Wnt targets in tissue isolated from the *Apc^Min/+^Lect2^+/+^* (*N* = 4) and *Apc^Min/+^Lect2^−/−^* (*N* = 4) cohorts. Whilst we were able to show the previously characterised induction of downstream Wnt targets during Wnt-driven tumorigenesis, the additional loss of Lect2 had no significant effect on the expression of key Wnt target genes (Figure 2E). Taken together this indicates that the homozygous loss of *Lect2* enhances tumour initiation and progression in the *Apc^Min/+^* model independently of an effect on the Wnt pathway.

### Loss of *Lect2* does not modify the activated-Wnt signature in the small intestine

As our data shows that *Lect2* modulates Wnt-driven tumorigenesis, but does not appear to directly effect Wnt-signalling in developed tumours we next addressed whether Lect2 may have a key role during the very early stages of Wnt-activation. We crossed the *Lect2^−/−^* allele onto a conditional *Apc* background [10] and analysed the effect of *Lect2* deficiency in the context of acute Wnt signalling following *Apc* loss. Cre activity was induced in both the *AhCre^+^Apc^fl/fl^Lect2^−/−^* and *AhCre*^+^*Apc^fl/fl^Lect2^+/+^* mice by intraperitoneal injection of β-naphthoflavone. Previous studies have demonstrated that three injections of 80 mg/kg of β-naphthoflavone in 24 hrs results in efficient (~100%) recombination of the *Apc* allele [11] in the mouse small intestine. Due to the previously reported [12] overt phenotype of induced *AhCre^+^Apc^fl/fl^Lect2^+/+^* mice both experimental and control cohorts were killed at day 5 post-induction (PI) and the tissues analysed.

**Figure 3:**
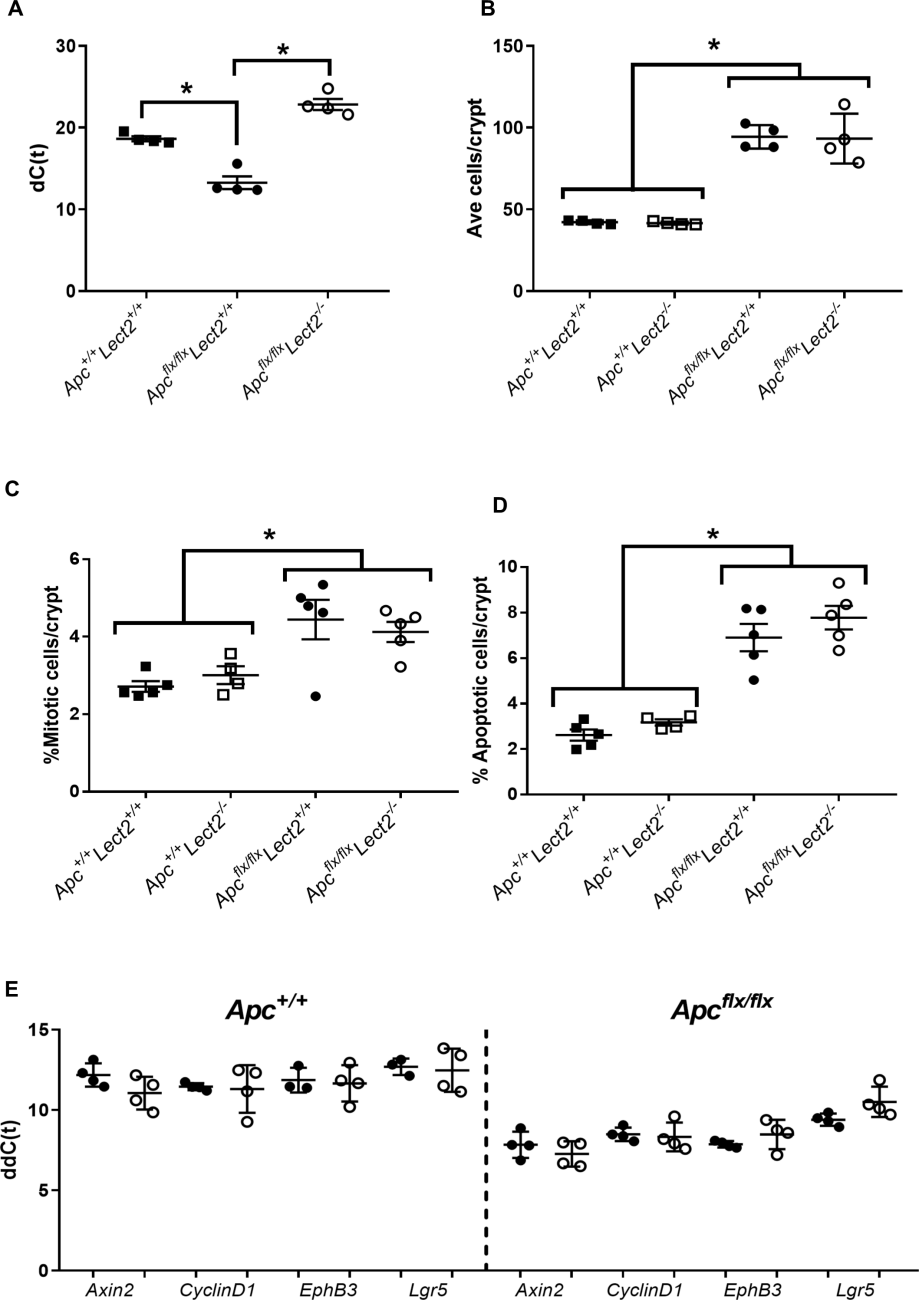
Combined loss of Apc and Lect2 in the mouse small intestine has no significant effect on the Apc homozygous phenotype (**A**) qRT-PCR analysis of *Lect2* expression 4 days following deletion of *Apc*. The data shown is in terms of the dC(t) values used to calculate the ddC(t) fold-change values. Analysis of crypt cell number (**B**), mitosis (**C**) and apoptosis (**D**) from H&E-stained histological sections of mouse small intestine indicated the alterations characteristic of acute *Apc* loss are unaffected by additional loss of *Lect2* (data shown are means ± S.D. of a minimum of 25 whole crypts from four independent experiments; Mann–Whitney ^*^P < 0.05). (**E**) qRT-PCR analysis of down-stream Wnt-targets from *Lect2*^+/+^ (●) and *Lect2*^−/−^ (○) *mice indicating Lect2* deficiency doesn't alter expression of Wnt target genes in the normal or *Apc*^fl/f^ small intestine.

qRT-PCR analysis of *Lect2* expression in the *AhCre+Apc^fl/fl^* confirmed Lect2 as a downstream target of Wnt signalling in the mouse small intestine (Mann–Whitney *P* = 0.0286; *N* = 4v4) and loss of *Lect2* expression in our experimental *AhCre^+^Apc^fl/fl^Lect2^−/−^* cohort (Mann–Whitney *P* = 0.0286; *N* = 4v4) (Figure 3A). To assess the phenotypic consequences of the deletion of both *Apc* and *Lect2* we analysed the crypt-villus structure. The number of cells per crypt were scored from haematoxylin and eosin-stained sections as described previously [12]. The data for the control mice (*AhCre^+^Apc^+/+^Lect2^+/+^, AhCre^+^Apc^fl/fl^Lect2^+/+^*; Figure 3B) at day 5 PI were consistent with previous data [12]. With a significant increase in crypt cell number between the wild-type and *AhCre^+^Apc^fl/fl^Lect2^+/+^* mice (*n* = 4, Mann–Whitney *U*-test *P* = 0.0304) (Figure 3B). Additional loss of the *Lect2* allele had no effect on either the wild-type (*N* = 4, Mann–Whitney *U*-test *P* = 0.3856) or the *Apc* homozygous phenotype (*N* = 4, Mann–Whitney *U*-test *P* = 0.6650). In addition to quantifying crypt cell number, apoptosis and mitosis were scored as described previously [12]. The data for the control mice (*AhCre^+^Apc^+/+^Lect2^+/+^, AhCre^+^Apc^fl/fl^Lect2^+/+^*; Figure 3C and 3D) at day 5 PI were consistent with previous reports [12] with a significant increase in mitosis and apoptosis between the *AhCre^+^Apc^fl/fl^Lect2^+/+^* (Mann–Whitney *U*-test, *P* = 0.0404) and wild-type mice, with no significant effect of *Lect2* deletion (Figure 3C and 3D). In addition we analysed the localisation of the Paneth cells in our mouse models. Paneth cell localisation is altered in the *Apc* homozygous mice [12] and previous studies using the *AhCre*^+^*Apc^fl/fl^Mbd2^−/−^* mice, where *Lect2* expression was shown to be upregulated, demonstrated a significant reduction in the mislocalisation of this cell population [4]. The expected mislocalisation of lysozyme-positive cells along the length of the aberrant crypt-villus axis was confirmed in the *Apc* homozygous mice, and *Lect2* loss had no further significant effect, either in the wild-type or *Apc* homozygous cohorts (Supplementary Figure 2A).

To further characterise the effect of *Lect2* on the Wnt signalling pathway in our short-term *Apc* homozygous model, we used qRT-PCR analysis to compare the expression of Wnt targets in tissue isolated from control and experimental cohorts. Whilst we demonstrated the previously characterised induction of downstream Wnt targets, due to *Apc* loss, the additional loss of Lect2 in either the *AhCre^+^Apc^+/+^Lect2^−/−^* or the *AhCre^+^Apc^fl/fl^Lect2^−/−^* mice had no significant effect on the expression of key Wnt targets in the small intestine (Figure 3E). In summary the loss of *Lect2* has no impact on cell homeostasis or Wnt activity in the intestine.

### *Lect2* has a role in regulating the circulating levels of key inflammatory markers

The above data argues that the observed modulation of Wnt-driven tumorigenesis by the absence of *Lect2* does not appear to be due to a cell autonomous role for Lect2 in regulating Wnt signaling. We have therefore explored other possible mechanisms for Lect2 action. Several studies have shown that, similar to other solid malignancies, colorectal tumours demonstrate inflammatory infiltration with multiple cell types that may have pro- or anti-tumorigenic role [13]. In addition, recent work by McClellan *et al.* [14] has demonstrated that the activation of key inflammatory mediators in a Wnt-activated CRC model produces a microenvironment that has an important impact upon tumoral development in the intestine. Lect2 is a chemokine and has been shown to have a key role in the inflammatory response in β-catenin-induced liver tumorigenesis where it is associated with a suppressive effect on inflammation-related cytokine production [3]. In order to analyse whether the tumour suppressor function of Lect2 in Wnt-driven tumorigenesis is due to its role in inflammation we measured key cytokine levels in our Wnt-activated models of CRC. We analysed serum from our *AhCre^+^Apc^+/+^* (*N* = 5), *AhCre^+^Apc^fl/+^Lect2^+/+^* (*N* = 5) and *AhCre^+^Apc^fl/+^Lect2^−/−^* (*N* = 5) at day 55 PI. The *AhCre^+^Apc^fl/+^* tumour model was used in our short-term cohorts as this model develops tumours at a similar latency to that of the *Apc^Min/+^* model, inducing *Apc* deletion at a fixed time point provides experimental consistency and *Apc* remains intact within the immune system. Samples from day 55 PI were analysed in order to investigate the effect of Wnt-activation and Lect2 loss on cytokine expression prior to tumour development.

**Figure 4:**
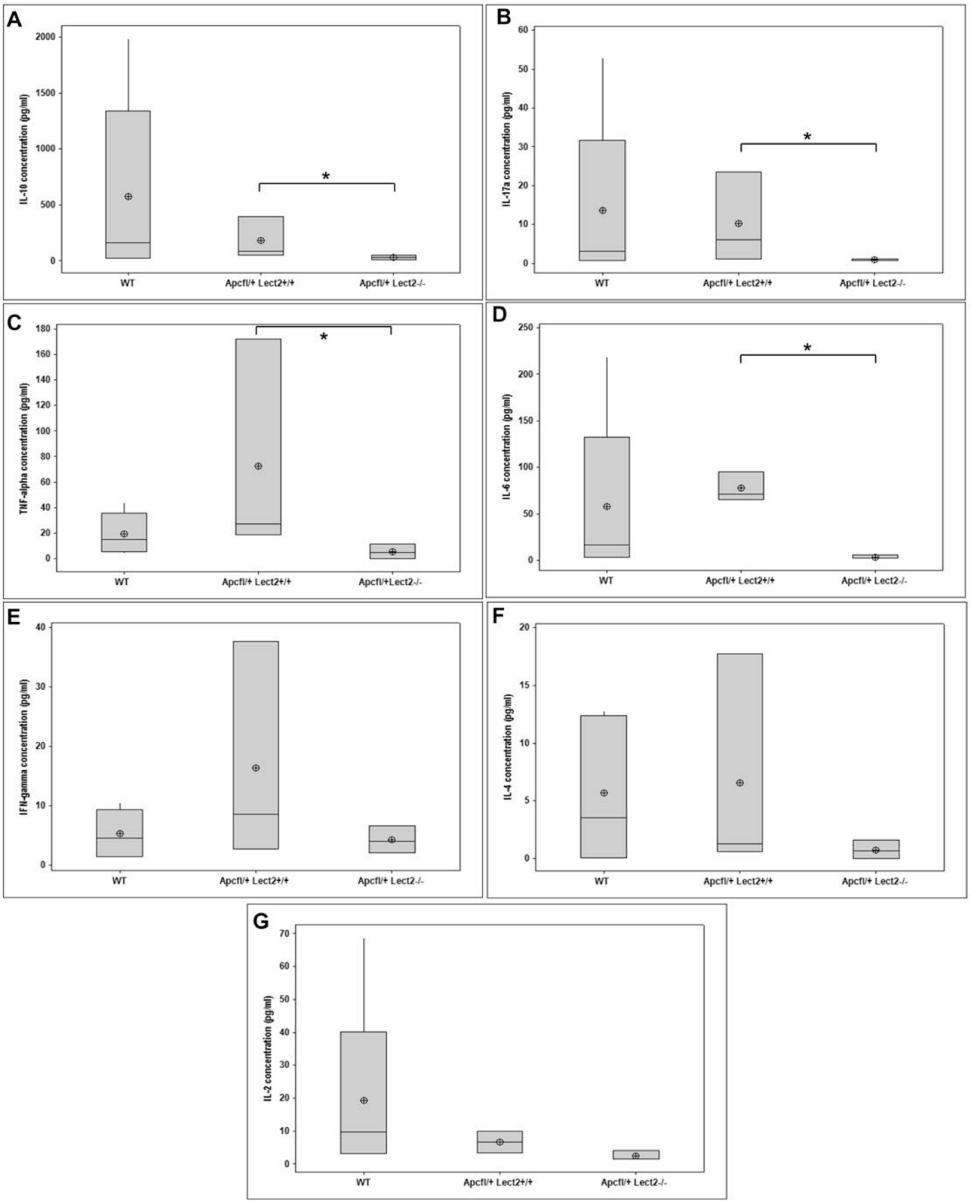
Loss of Lect2 alters the circulating concentration of key cytokines ELISA analysis indicating a general decrease in circulating cytokines in serum from WT, *AhcreApc^+/fl^* and *AhcreApc^+/fl^Lect2^−/−^* at 55 d.p.i. for IL-10 (**A**), IL-17 (**B**), TNF-alpha (**C**), IL-6 (**D**), Ifng (**E**), IL-4 (**F**), and IL-2 (**G**) (data shown are means ± S.D. of a minimum of four independent experiments (all *N* = 5). ^*^Mann–Whitney ^*^P < 0.05).

At day 55 PI, *Apc* heterozygosity did not alter the circulating levels of several cytokines compared to wild-type mice (Figure 4). In contrast, *Lect2* deficiency in this model resulted in a significant decrease in IL-10 (*AhCre^+^Apc^fl/+^Lect2^−/−^* versus *AhCre^+^Apc^fl/+^Lect2^+/+^*; median concentration 31.75 pg/ml versus 87.49 pg/ml; *P* = 0.0404; Figure 4A), IL-17a (*AhCre^+^Apc^fl/+^Lect2^−/−^* versus *AhCre^+^Apc^fl/+^Lect2^+/+^*; median concentration 1.08 pg/ml versus 6.05 pg/ml; *P* = 0.0404; Figure 4B), TNF-α (*AhCre^+^Apc^fl/+^Lect2^−/−^* versus *AhCre^+^Apc^fl/+^Lect2^+/+^*; median concentration 4.74 pg/ml versus 27.34 pg/ml; *P* = 0.0404; Figure 4C) and IL-6 (*AhCre^+^Apc^fl/+^Lect2^−/−^* versus *AhCre^+^Apc^fl/+^Lect2^+/+^*; median concentration 2.51 pg/ml versus 71.19 pg/ml; *P* = 0.0404; Figure 4D). In addition several other key cytokines showed trends towards a decrease in their levels although this was not significant, such as IFN-γ (*AhCre^+^Apc^fl/+^Lect2^−/−^* versus *AhCre^+^Apc^fl/+^Lect2^+/+^*; median concentration 4.11 pg/ml versus 8.68 pg/ml; *P* = 0.1914; Figure 4E), IL-4 (*AhCre^+^Apc^fl/+^Lect2^−/−^* versus *AhCre^+^Apc^fl/+^Lect2^+/+^*; median concentration 0.66 pg/ml versus 1.26 pg/ml; *P* = 0.3313; Figure 4F) and IL-2 (*AhCre^+^Apc^fl/+^Lect2^−/−^* versus *AhCre^+^Apc^fl/+^Lect2^+/+^*; median concentration 1.62 pg/ml versus 6.73 pg/ml; *P* = 0.0952; Figure 4G). This trend towards a global reduction cytokine levels indicates that *Lect2* plays a role in driving or maintaining the inflammatory response that occurs in the presence of Wnt driven intestinal tumours.

### *Lect2* deficiency increases the proportion of CD4+ T cells in the spleen

**Figure 5:**
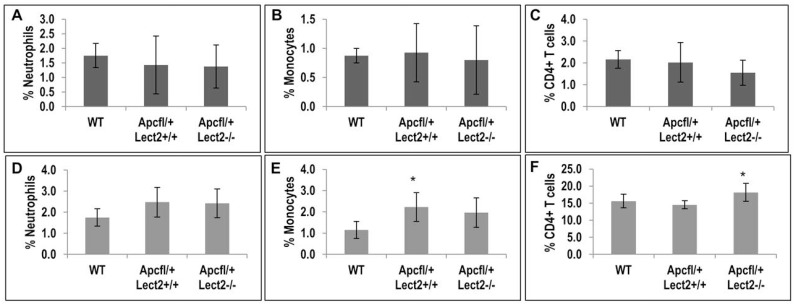
Loss of Lect2 significantly up-regulates the CD4+ T cell population (**A**–**F**) FACS analysis of mononuclear cells in liver and spleen from *AhCre^+^Apc^fl/+^Lect2^+/+^* and AhCre^+^Apc^fl/+^Lect2^−/−^ at 55d.p.i. The graphs represent the percentage of cells gated on CD45+ cells in either the liver (A–C) or the spleen (D–F) (data shown are means ± S.D. of a minimum of four independent experiment, all *N* = 5 Mann–Whitney ^*^P < 0.05).

Previous studies have identified a key role for Lect2 in the regulation of specific sub-populations of inflammatory cells and this function was key to the tumour suppressor role of Lect2 in β-catenin-activated HCC [7]. To evaluate whether the ability of Lect2 to alter the cytokine levels was related to an alteration in immune cell populations we measured the proportions of immune cells in the liver and the spleen at day 55 PI by FACS. Analysis of proportions of neutrophils, monocytes and total mononuclear cells, in both these models indicated no significant changes due to *Lect2* loss (Figure 5A, 5B, 5D and 5E and Supplementary Figure 3A and 3C). However, we did show a significant increase in the proportion of monocytes in the spleen of *AhCre^+^Apc^fl/+^Lect2^+/+^* mice compared to wild-type (2.2% versus 1.2%; two-tailed *t* test on Log10 transformed data, *P* = 0.031; Figure 5E). Further analysis of the proportions of NK cells, NKT cells, conventional CD3+ T cells and CD4+ NKT cells in both the spleen and the liver and found no significant differences in either model (Supplementary Figure 3A–3J). However, the analysis of CD4+T cells in the spleen of our *AhCre^+^Apc^fl/+^Lect2^−/−^* mice identified a small but significant increase compared to the *AhCre^+^Apc^fl/+^Lect2^+/+^* model (18.2% versus 15.6%; two-tailed *t* test on Log10 transformed data, *P* = 0.010; Figure 5F). Although no such difference was observed in the liver (Figure 5C). This data combined with the cytokine alterations (Figure 3) identified potentially indicates a role for *Lect2* in maintaining a tumour suppressive immune intestinal microenvironment.

### *Lect2* deficiency influences *Treg* cells in the loss of Lect2 influences the

**Figure 6:**
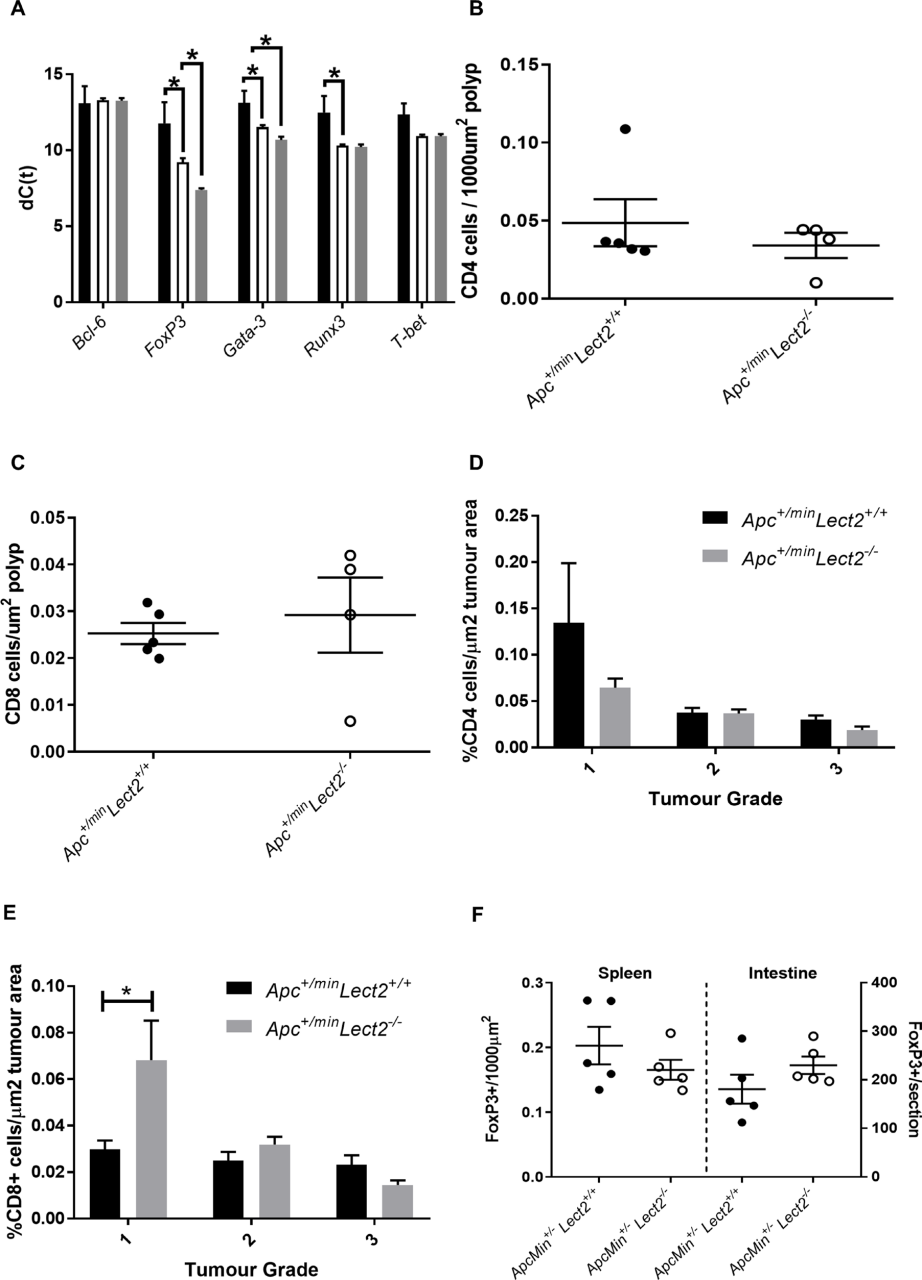
Lect2 regulates the expression of lineage-specification factors in the spleen (**A**) qRT-PCR analysis of tissue expression of key transcription factors in *AhCre^+^Apc^+/flx^Lect2^+/+^* (white bars) and *AhCre^+^Apc^+/flx^Lect2^−/−^* (grey bars) compared to the normal splenic tissue from *AhCre^+^Apc^+/+^Lect2^+/+^* mice (black bars) (data shown is in terms of dC(t) values used to calculate ddC(t) fold change) (Mann–Whitney ^*^P < 0.05. Quantification of CD4+ (**B**) and CD8+ (**C**) cells in small intestinal polyps. Quantification of CD4+ (**D**) and CD8+(**E**) cells indicates an increase in the density of CD8+ cells inT1 stage tumours from *Apc^+/min^Lect2^−/−^* mice compared to control *Apc^+/min^* mice (all *N* = 4; ^*^Mann–Whitney *P* > 0.05). Quantification of CD3+FoxP3+ Treg cells in spleen and small intestine (**F**).

To understand the relationship between the widespread deregulation of circulating cytokines and proportional increase in splenic CD4+ T cells in the *AhCre^+^Apc^fl/+^Lect2^−/−^* mice we analysed the expression of key transcription factors that regulate cytokine expression and T cell differentiation. Several master transcription factors have been identified which regulate T cell fate and cytokine expression during tumourigenesis [15, 16] and the activity of these regulators is primarily determined by their expression levels. Therefore, using qRT-PCR we analysed the expression of *Gata-3*, *T-bet*, *FoxP3*, *Bcl-6* and *Runx3*, all of which function as part of a network to regulate the inflammatory response. The expression of these transcription factors was analysed in the spleens of both the *AhCre^+^Apc^fl/+^Lect2^+/+^* (*N* = 4) and the *AhCre^+^Apc^fl/+^Lect2^−/−^* (*N* = 4) cohorts at day 55 PI (Figure 6A). Interestingly *Gata-3* (2.99-fold up-regulation; *P* = 0.0428), *FoxP3* (5.86-fold up-regulation; *P* = 0.0214) and *RunX3* (4.48-fold up-regulation; *P* = 0.0142) all showed a significant increase in the *AhCre^+^Apc^fl/+^Lect2^+/+^* mice compared to wild-type (Figure 6A), and both *GATA-3* and *FoxP3* were further increased in the *AhCre^+^Apc^fl/+^Lect2^−/−^* model (1.8- and 3.5-fold respectively; *P* = 0.0051 and *P* = 0.0025). The expression of both *T-bet* and *Bcl-6* showed no significant change in either model. The *Lect2* dependent increase in *Gata-3 and FoxP3* indicated a role for the immune suppressive CD4+ Treg cell population [17]. *Foxp3* is a marker of CD4+ immunosuppressive Treg cells, which could suppress an anti-cancer immune response and are associated with tumour progression [18]. To address whether the altered expression of the lineage factors was reflected in the numbers of immune cells within the tumours we quantified the numbers of CD4+, CD8+ and Treg cells in splenic tissue and intestinal tissue using immunohistochemical staining (number of was based on the average number of cells in 5 sequential longitudinal sections taken every 100 μm). Immunohistochemical analysis of the groups indicated no overall difference density of CD4+ or CD8+ between polyps from *Apc^Min/+^Lect2^+/+^* and *Apc^Min/+^Lect2^−/−^* cohorts (Figure 6B and 6C). The major alteration identified was a significant increase in the density of CD8+ cells, but not CD4+, in T1 tumours from *Apc^Min/+^Lect2^−/−^* mice, the stage significantly decreased in this model (Figure 6D and 6E). Subsequent analysis of CD4+FoxP3+ Treg cells indicated no significant difference in the spleen and a non-significant trend towards an increase in the intestine (Figure 6F; *Apc^Min/+^Lect2^−/−^* (*N* = 4) and *Apc^Min/+^Lect2^+/+^*(*N* = 5)). Thus, the increase in *FoxP3* expression observed in the spleen is likely due to it being upregulated within the *in situ* Treg cells. While not conclusive, the trend towards an increased number of Tregs in the intestine supports the premise that the increase in number and progression of Wnt driven tumours in the *Apc^min/+^Lect2^−/−^* tumour progression is related to a deregulation of the immune response to intestinal tumours.

## DISCUSSION

Whilst the inhibition of the Wnt signalling pathway is thought to have clear therapeutic potential in the treatment of CRC, the complexity of this signalling cascade has hindered the development of targeted treatment for this disease. Therefore, a clearer understanding of this signalling pathway, through the identification of key regulatory proteins and important downstream targets of β-catenin will significantly improve the development of treatments for Wnt-driven tumorigenesis. Lect2 is a downstream target of the Wnt pathway and has a key role in inflammation, although its precise mechanism of action *in vivo* is still unclear. In the liver Lect2 has been shown to have a protective anti-inflammatory role in β-catenin-induced tumorigenesis [3] and the role of Lect2 as a tumour suppressor in human HCC has been firmly established [19]. Lect2 has also been shown to be a downstream target of the Wnt pathway in the intestine and has been hypothesised to have a protective role during Wnt-driven tumorigenesis [4].

Our previous studies investigating the role of the methyl binding protein Mbd2 in Wnt-driven CRC identified Lect2 as a downstream target of the Wnt pathway in the intestine and demonstrated a key role for Lect2 as a Wnt inhibitor [4]. To confirm and further investigate the function of Lect2 in Wnt-driven CRC, we homozygously deleted Lect2 in the *Apc^Min/+^* mouse model. Using this model, we identified a novel role for Lect2 as a tumour suppressor in Wnt-driven intestinal tumorigenesis. Loss of Lect2 resulted in a significant decrease in survival in our *Apc^Min/+^* mouse model, which was associated with a significant increase in adenoma number in the small intestine. In addition, the number of advanced tumour lesions was increased, despite the 30% reduction in the longevity of the mice, indicating that the homozygous loss of *Lect2* enhances both Wnt-activated tumour initiation and progression, identifying a clear protective role for Lect2 in intestinal tumorigenesis. However, our data indicating the loss of *Lect2* had no impact on the phenotype of the *Apc^+/min^Mbd2^−/−^* mice suggests that epigenetic silencing of *Lect2* doesn't play a key role in intestinal tumour initiation but is more relevant to inflammation and tumour progression [20].

As Lect2 was previously indicated to function as a Wnt repressor we hypothesised that the tumour suppressor role of Lect2 may be due to a protective inhibitory function in the initiating stages of Wnt deregulation. Therefore, to investigate the effects of Lect2 loss on the Wnt signalling pathway we used an early-stage Wnt-activated model to characterise the effect of Lect2 loss on the initial stages of aberrant Wnt signalling in the mouse small intestine. Our results demonstrate that whilst Lect2 is a downstream target of the Wnt pathway, homozygous loss of this tumour suppressor had no effect on the activated Wnt signature, indicating that the protective role of Lect2 may be independent of Wnt signalling in the mouse intestine.

In addition to its potential role as a Wnt inhibitor Lect2 has been shown to have a key role in inflammation. Lect2 has a protective anti-inflammatory role in arthritis and in the liver Lect2 regulates the homeostasis of NKT cells and also the expression of IL-4 and IFN-γ [5]. Furthermore, Lect2 has been shown to be a mediator of the β-catenin inflammatory response during hepatocellular carcinoma and loss of this tumour suppressor results an increase in tumorigenesis [3]. Therefore, we investigated whether loss of Lect2 had an effect on the inflammatory response during colorectal tumorigenesis.

Our results demonstrated that the loss of Lect2 in our Wnt-activated model significantly altered the levels of circulating cytokines. The effect was widespread with levels of both pro- and anti-inflammatory cytokines being suppressed upon the homozygous loss of *Lect2*, which was further seen in the local inflammatory environment of key tissues such as the spleen and the liver, although to a lesser extent in the small and large intestine. A similarly altered cytokine secretion profile in response to the loss of Lect2 has been previously shown in the liver and this altered profile in *Lect2^−/−^* mice was shown to be associated with an alteration in the profile of infiltrating immune cells [5, 21]. Thus, it seem that *Lect2* loss is well tolerated but alters systemic inflammatory responses when aberrant oncogenic Wnt activation occurs in target organs. This is possibly due to impaired barrier function with the host microbiome, in accordance with other data indicating that Ifng and TNF-a production in NK and NKT cells is lower in *Lect2^−/−^* mice exposed to bacterial LPS [21]. It is well established that genetic alterations involved in driving tumorigenesis activate an inflammatory program that has a significant impact on tumour development [22] and several studies have highlighted the role of inflammation in CRC. However, the exact role that each cytokine plays is clouded by conflicting data as to whether they are pro- or anti-tumourigenic (reviewed by Mager *et al* [23] and Chen and Zhou [24]. For example IL-10, which is reduced in the *Apc*^*+/*flx^Lect2^−/−^ mice, has been shown to be a key cytokine which when produced by Treg cells that can reduce tumour burden in the *Apc^+/min^* model [25, 26]. Despite the Treg marker *FoxP3* being increased in our models there was no increase in the number of Treg cells indicating that either *Foxp3* upregulation inhibits they’re ability to produce Il-10 or it is due to dysfunction in other Il-10 producing cells such as macrophages, mast cells eosinophils and dendritic cells [27]. The Il-10 reduction is consistent with other studies that have shown that T cell-restricted ablation of IL-10 increased the number of polyps by promoting the accumulation of microbes and eosinophils [28] and IL-10 deficient mice are more susceptible to spontaneous intestinal tumour development [29]. Therefore, in order to further investigate the role of Lect2 in the immune response during Wnt-driven tumorigenesis we characterised the subpopulations of inflammatory cells in both the spleen and liver. Our results show that the loss of Lect2 leads to a significant increase in the proportion of the CD4^+^ subpopulation of T cells in the spleen.

CD4^+^ T cells carry out multiple functions, ranging from activation of the cells of the innate immune system, B-lymphocytes, cytotoxic T cells, as well as non-immune cells, and they also play critical roles in the suppression of the immune reaction. Subsets of CD4^+^ T cells include the classical Th1 and Th2 cells and differentiation of the different lineages depends on a complex network of specific cytokine signalling and lineage specific transcription factors. An imbalance of Th1 and Th2 cells is thought to be responsible for both the occurrence and also the progression of several diseases, and patients with advanced cancer often have impaired cell-mediated immunity associated with a switch from Th1 to Th2 [30–32]. Work by Anson *et al*. [3] demonstrated that loss of Lect2 significantly altered the inflammatory microenvironment by shifting the balance towards a Th2 pro-tumorigenic inflammatory program, which allowed tumour growth and progression.

As the presence of CD4+ cells can be anti- or pro-tumourigenic depending on the lineages present [33] we therefore investigated whether loss of Lect2 resulted in an alteration in the expression of key transcription factors required for the differential development of the antigen-activated CD4^+^ T cells. Our results demonstrate that loss of Lect2 significantly increased the expression of *GATA-3* and *FoxP3* in the spleen. GATA-3 is a Th2 master regulator that is critical for the development of CD4+ Th2 cells, and FoxP3 expression is required for the generation of immune-suppressive CD4+CD25+ regulatory T cells (Tregs). GATA-3 also positively regulates FoxP3 expression to regulate Treg cell function [34] and the Treg cell lineage is thought to inhibit the protective anticancer inflammatory response [18]. While we failed to see an increase in the number of Treg cells in our models the increase in FoxP3 expression may reflect an increase in their turnover or regulatory abilities. As it has been previously shown that mast cells, which accumulate in *Apc^+/min^* polyps and human colorectal cancer are linked to progressive polyp growth [35], as they interact with Treg cells to generate potently immune suppressive but pro-inflammatory FoxP3+ cells that are characterised by a reduction in IL-10 production [36], a feature observed in our model. In addition, outside the haemopoietic system, both FoxP3 and GATA-3 have various roles in tumour development which may be pro- or anti-tumorigenic, depending on the tumour type [37, 38].

In conclusion, our data demonstrate a novel role for Lect2 as a tumour suppressor during Wnt-driven intestinal tumorigenesis. Whilst this role is independent of a direct effect on the Wnt pathway, our results indicate that Lect2 functions as a mediator of the inflammatory response during Wnt activation in the intestine and the loss of this chemokine alters the balance of both pro and anti-inflammatory cytokines and key regulators of T cell lineages. This alteration in T cell regulators may alter CD4^+^ T cell subsets and disrupt the immune environment, promoting tumour growth, although further studies are required to confirm this.

## MATERIALS AND METHODS

### Experimental mice

Animals were maintained on an outbred background, housed in a standard facility and all experimental procedures were performed in accordance with institutional animal care and ARRIVE guidelines in compliance with UK Home Office regulations. In brief, mice were maintained in conventional open top cages on dust free bedding (IPS Ltd) under a 12 hr light cycle, with RM3(E) diet (Special Diet Services UK) provided for nutritional support. To enrich the environment, sunflower seeds (at weaning only, LBS Ltd), nestlets (IPS Ltd), disposable envirotubes (IPS Ltd) and small chewsticks (Labdiet–IPS Ltd) were provided. Mice carrying the targeted *Lect2* allele were kindly supplied by Dr Satoshi Yamagoe [5]. Experimental mice were genotyped as previously described for the targeted *Lect2* allele [5], *Apc* allele [10], the *Apc^Min/+^* allele [39], the *Rosa26R* allele [40] and the *AhCre* transgene [11]. Cre activity was induced in control and experimental mice by 3 consecutive intraperitoneal (i.p.) injections of 80 mg/kg β-naphthoflavone (Sigma, UK) in 24 h. Mixed sex control (litter mates) and experimental mice were used for timepoint (*N* > 4) or survival (*N* > 15) experiments. Prior to proliferation analysis selected animals were injected with 100 μg/kg Bromo-deoxyuridine (Sigma, UK) and culled at indicated time points after labelling.

### Histology and immunohistochemistry

Intestinal tissue was fixed, processed and haematoxylin and eosin stained as described previously [41]. The following antibodies were used for immunohistochemistry: anti-Cd4(1:100; eBioscience), anti-Cd8 (1:200; eBioscience) anti-Caspase 3 (1:750; R&D systems), anti-β-catenin (1/50; Becton Dickinson), anti-Ki67 (1:200; Vector Labs) and mouse anti-BrdU (1:100; Becton Dickinson). Staining for Treg cells was performed on a Ventana (Roche) Discovery Ultra Autostainer (Serial number 313108) using Antigen Retrieval CC1 buffer (Ventana) for 48 minutes at 95° C and an Anti-mouse/rat FoxP3 (1/25; eBioscience) in discovery antibody diluent (Roche).

### Cellular analysis

Cellular analysis was performed on >25 whole crypts from at least three mice of each genotype. Apoptotic and mitotic index were scored from haematoxylin-and-eosin-stained sections as previously described (36). The cells between the base of the crypt and the junction with the villus was designated as the proliferative zone. For migration analysis mice of 60–80 days of age were given an IP injection of BrDU 2 hours or 24 hours prior to culling and dissection. Immunohistochemical analysis for BrDU incorporation was performed on formalin fixed small intestinal rolls, and the number BrDU positive cells and their location (with 0 being the bottom of the crypt) was measured on 50 half-crypts per mouse, minimum of 4 mice. Statistical analysis of the cumulative frequency of positive cells was performed using a two-tailed Kolmogorov-Smirnov test, on graphs *P* values are indicated as follows: ^*^P < 0.05; ^**^P < 0.01; ^***^P < 0.001.

### Quantitative real-time RT-PCR

Total RNA was isolated from tissues using a standard Trizol protocol and DNAse treatment was carried out using the Turbo DNA-free kit (Life Technologies Ltd., UK). One microgram of RNA was reverse transcribed using Superscript III according to the manufacturer's protocol (Life Technologies Ltd., UK). Gene expression analysis was carried out using either TaqMan Universal PCR mastermix or Fast Sybr green mastermix according to the manufacturer's protocol (Life technologies Ltd., UK). The primer sequences used in the PCR reactions available upon request. Primers and TaqMan probes specific for *Lgr5, IL-4, IFN-gamma, TNF-alpha, FoxP3* and *B-actin* were obtained from Taqman gene expression assays (Life Technologies Ltd., UK). Data analysis was carried out using StepOne™ Software v2.2.2 (Life Technologies Ltd., UK). Relative expression levels of target genes were calculated using the comparative cycle threshold (Ct) method as described previously [42]. The values for *β-actin* were used to normalize the gene expression data. The gene expression levels in intestinal tumours relative to the control intestinal normal tissues were calculated using the following formulae: ΔΔCt = ΔCt test−ΔCt control, fold change = 2−ΔΔCt [43].

### Cytokine ELISA

The levels of serum IFN-γ, TNF-α, IL-10, IL-6, IL-4, and IL-17a were quantified using the BD Cytometric Bead Array mouse Th1/Th2/Th17 Cytokine kit (BD Pharmingen, Oxford, UK).

### Flow cytometric analysis

Single cell suspensions from the liver and the spleen were incubated with monoclonal antibodies against cell surface markers (BD Pharmingen, Oxford, UK). APC- and FITC- conjugated antibodies specific for CD3 (145-2C11), CD4 (RM4-5), NK1.1 (PK136), CD11b (M1/70), Gr-1 (RB6-8C5), Mac-1/Cd11b (M1/70) were used for flow cytometric analysis. Resident monocytes were identified as CD11b+Gr-1- and neutrophils as CD11b+Gr-1+ cells with an intermediate SSC profile [44].

## SUPPLEMENTARY MATERIALS FIGURES


