# Supplementary material for: Lect2 deficiency is characterised by altered cytokine levels and promotion of intestinal tumourigenesis
Source: Oncotarget. 2018 Nov 23;9(92):36430–43. doi: 10.18632/oncotarget.26335 (PMC6284865; doi:10.18632/oncotarget.26335)
Supplement: Supplementary file 1 [file oncotarget-09-36430-s001.pdf]

Lect2 deficiency is characterised by altered cytokine levels and promotion of intestinal tumourigenesis

SUPPLEMENTARY MATERIALS

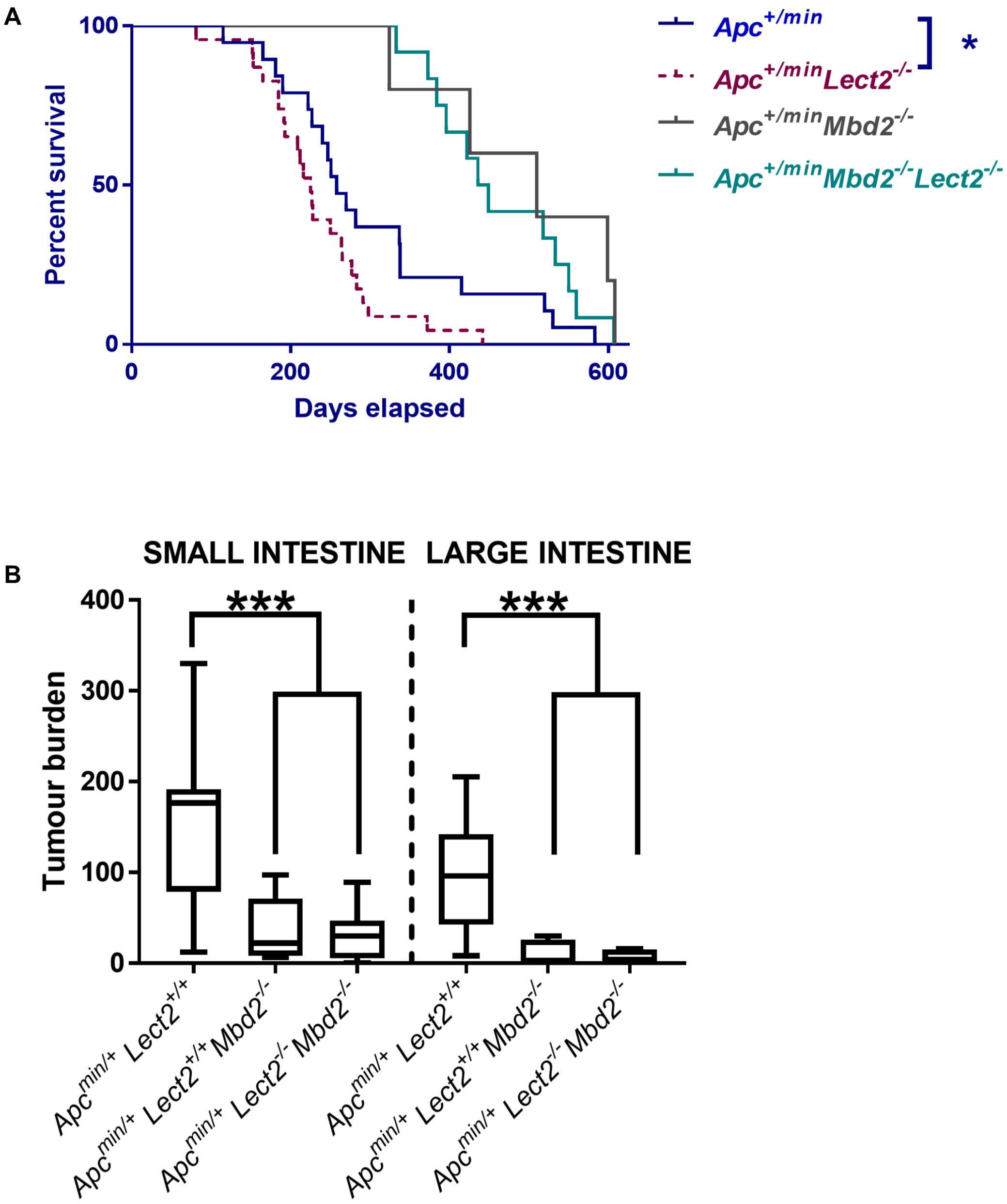

Supplementary Figure 1: The intestinal tumour resistant *Apc*$^{Min/+}$*Mbd2*$^{-/-}$ mice are unaffected by the additional loss of Lect2. Survival curve indicating no difference in lifespan (**A**) or tumour burden (**B**) at death between *Apc*$^{Min/+}$*Mbd2*$^{-/-}$ ($N = 6$) and *Apc*$^{Min/+}$*Mbd2*$^{-/-}$*Lect2*$^{-/-}$ ($N = 14$) mice (***Mann–Whitney $P < 0.001$).

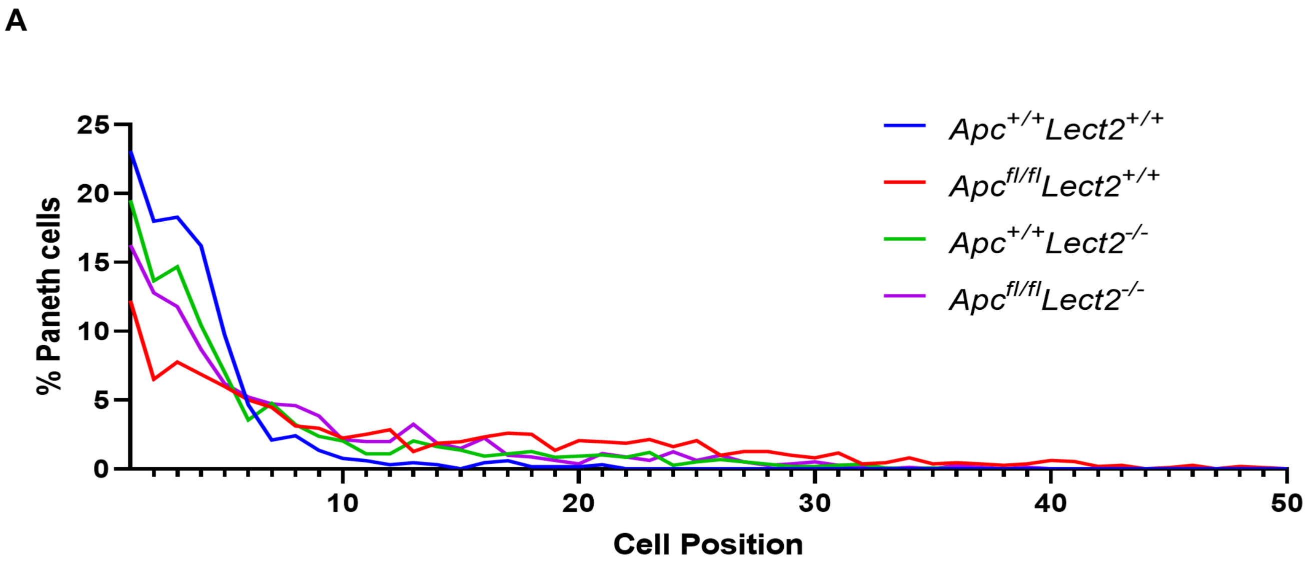

Supplementary Figure 2: No effect of Lect2 deficiency on Paneth cell position in the Wnt activated intestine. Lect2 deficiency doesn't alter Paneth cell localization (**A**) in the in the normal or Apc deficient small intestine

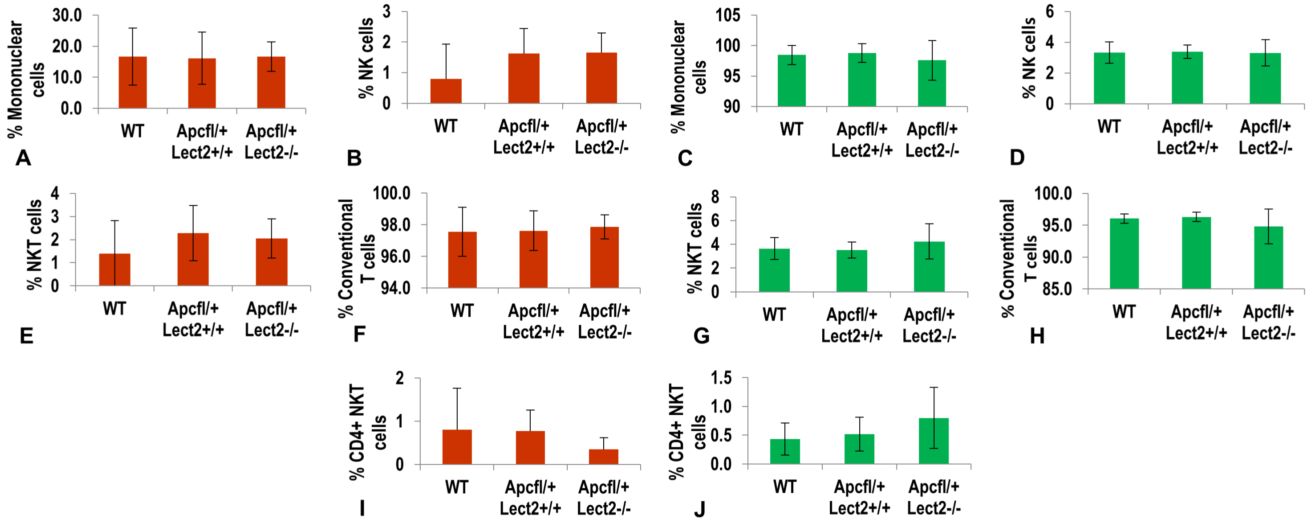

Supplementary Figure 3: Analysis of lymphocyte populations in *Lect2* deficient mice. Flow cytometry analysis indicated no alteration to the populations of cells indicated due to the loss of *Lect2* in *Apc*$^{+/fl}$ intestine at 55 d.p.i.. Panel indicating cells as a percentage of total leukocytes from liver (red columns) and spleen (green columns) for mononuclear (**A** and **C**), natural killer (NK; **B** and **D**), natural killer T cells (NKT; **E** and **G**), conventional T cells (**F** and **H**) and CD4+NKT cells (**I** and **J**).
